# Micro-RNA expression data from common carp brain cells infected by Cyvirus cyprinidallo3 at permissive and non-permissive temperatures

**DOI:** 10.1016/j.dib.2025.111800

**Published:** 2025-06-18

**Authors:** Olivier Rué, Nurul Novelia Fuandila, Saliha Hammoumi, Christine Gaspin, Jean-Christophe Avarre

**Affiliations:** a Université Paris-Saclay, INRAE, MaIAGE, Jouy-en-Josas, France; b Université Paris-Saclay, INRAE, BioinfOmics, MIGALE bioinformatics facility, Jouy-en-Josas, France; c ISEM, University of Montpellier, CNRS, IRD, Montpellier, France; d INRAE, BioinfOmics, GenoToul Bioinformatics Facility, Université de Toulouse, Castanet-Tolosan, France; e INRAE, MIAT, Université de Toulouse, Castanet-Tolosan, France

**Keywords:** CyHV-3, Cell infection, Microtranscriptome, Expression kinetics

## Abstract

MicroRNAs (miRNAs) are small non-coding RNAs capable of altering gene expression. In viruses, miRNAs may significantly influence the interaction between the virus and its host. Among all the viruses that have been reported to encode miRNAs, those belonging to the *Herpesvirales* order encode the largest number of pre-miRNAs. *Cyvirus cyprinidallo3* (CyHV-3) is a member of the *Alloherpesviridae* family and is recognised as a significant threat for the common carp and ornamental koi aquaculture.

The present study aimed to identify miRNAs involved in the lytic cycle of CyHV-3 and monitor their expression from 1 h to 10 days post-infection (dpi), at two different temperatures: a permissive temperature (22 °C) and a non-permissive temperature (30 °C) (Fig. 1). This is the first study that describes the expression kinetics of predicted miRNAs from CyHV-3-infected carp cells.

Alignment of reads against a CyHV-3 reference genome led to the identification of 81 putative pre-miRNAs. Although this data mainly focused on CyHV-3 miRNAs, an alignment of reads against the *Cyprinus carpio* genome led to the identification of 3025 miRNA that could be annotated.

Of note, the 7 miRNAs previously identified in CyHV-3 were retrieved among the 81 putative pre-miRNAs found here. The number of putative miRNAs identified is far higher than in the two previous studies, probably because of the high number of reads obtained across the 36 individual samples that represented all stages of viral infection.

A constant increase in the expression of virus-predicted miRNAs was observed along the viral infection at 22 °C, with a maximum at 6 dpi (Fig. 2). Interestingly, cytopathic effects started to be observed after 3 dpi, and all the cells were lysed after 10 days. In contrast, no cyctopathic effects were recorded at 30 °C; at this temperature, no difference could be observed between samples in terms of miRNA expression (Fig. 2).

Specifications Table

**Table:** 

Subject	Aquatic virology
Specific subject area	Microtanscriptomics
Type of data	FASTQ files of small RNA-seq raw and cleaned data; text files; FASTA files; GFF3 files
Data collection	A cell culture was inoculated with a CyHV-3 strain; infected cells were harvested from 1 hour to 10 days post-infection; miRNAs were extracted and each sample was sequenced using Illumina HiSeq2000, in a 50-bp single end format (36 datasets).
Data source location	Institut des Sciences de l’Evolution de Montpellier, France
Data accessibility	Repository names: NCBI, EBI Data identification number: PRJNA287728 (from SRX25019289 to SRX25019324) Direct URL to data: https://www.ebi.ac.uk/ena/browser/view/PRJNA287728 https://www.ncbi.nlm.nih.gov/bioproject/PRJNA287728
Related research article	None

## 1 Value of the Data


 

• Although CyHV-3 has the longest genome of all herpes viruses, very little is known about its genomic and transcriptomic characteristics. This dataset is the largest ever generated on the microtanscriptome of CyHV-3, both in terms of sequencing depth and of infection kinetics.
• In addition to providing information on the sequences and potential annotations of all identified pre-miRNAs, these data also give insight into the expression kinetics of the pre-miRNAs that are transcribed during a complete CyHV-3 lytic cycle.
• The high homogeneity in the read counts between the two replicates of each sample strengthens the reliability of these data.

**Fig. 1:**
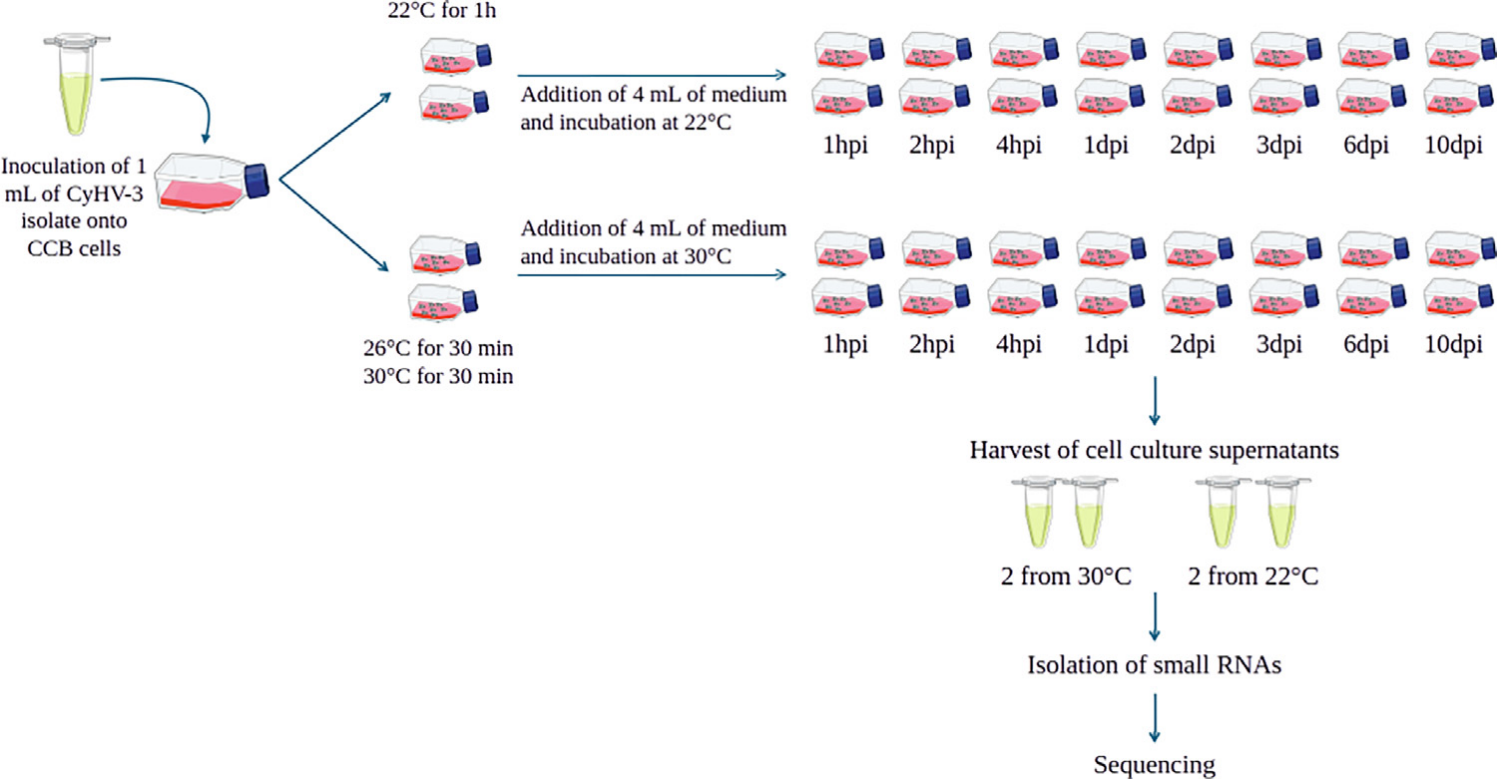
Schematic representation of the experimental design.

**Fig. 2:**
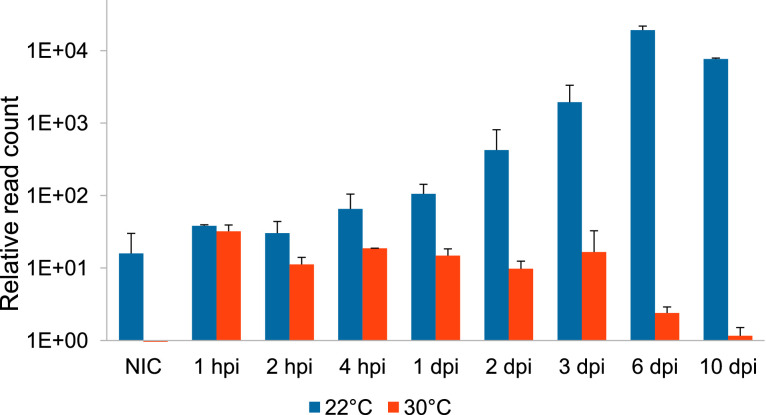
Kinetics of putative CyHV-3 miRNA abundance (mean of the two replicates ± SD) along viral infection at the two temperatures. For each sample, the abundance was calculated by dividing the total number of putative miRNA reads that aligned with CyHV-3 genome by the total number of reads, and multiplied by 10^6^. NIC: non-infected control; hpi: hours post-infection; dpi: days post-infection.

## 2 Background

So far, two aquatic herpes viruses have been reported to express miRNAs: *Cyvirus cyprinidallo3* [1,2] and *Cyvirus cyprinidallo2* [3]. In CyHV-3, 7 putative pre-miRNAs were identified, and most of them were confirmed by either Northern-blot or qPCR analysis [1,2]. However, these studies did not provide any details regarding the expression kinetics of these miRNAs during a lytic cycle. The present work was designed to monitor CyHV-3 miRNA expression during a complete infection cycle, up to 10 days after initial infection.

## 3 Data Description

Raw sequences (FASTQ files) were deposited in the public Sequence Read Archive (SRA) repository and can be accessed under the bioproject PRJNA287728, and the specific biosample SAMN42006672.

**Fig. 3:**
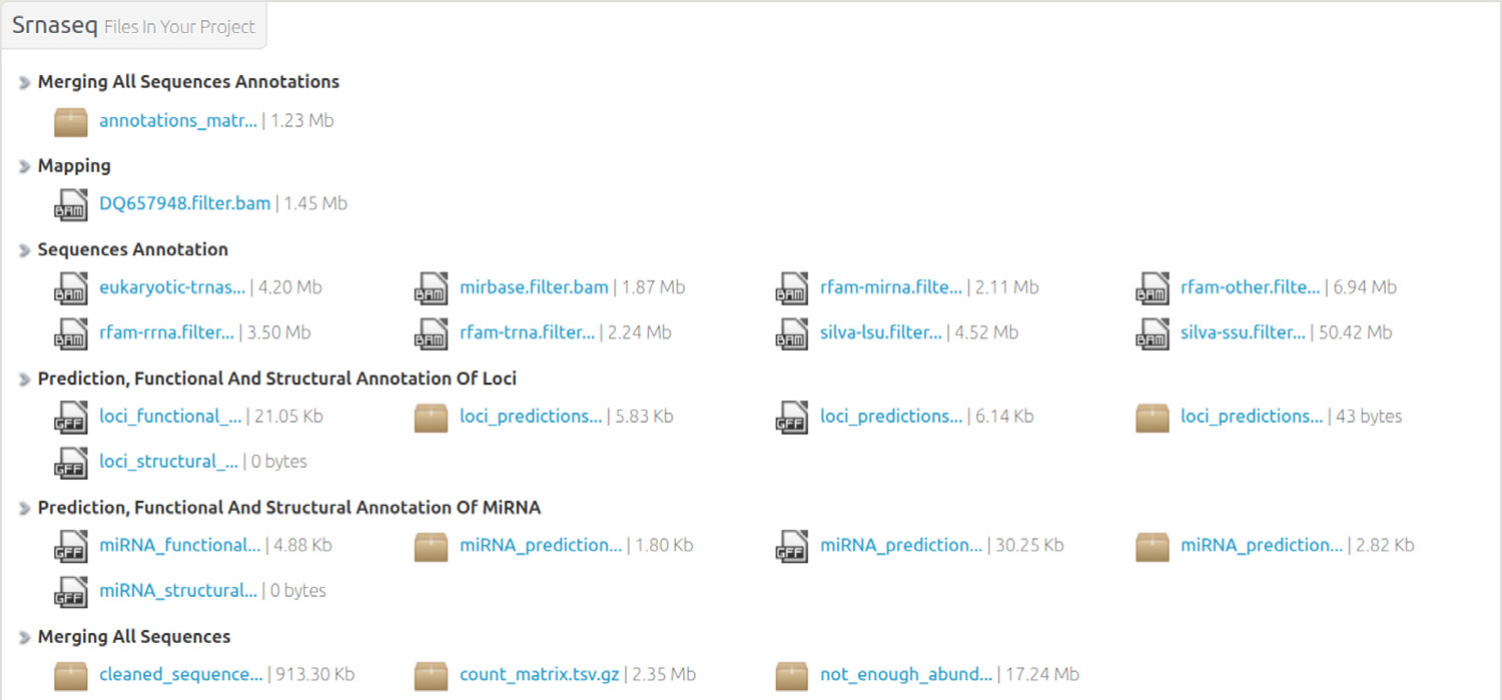
Available files for download.

Numerous files related to advanced data analyses are available through a web reporting page (http://ngspipelines2.toulouse.inra.fr:9073, download page after species selection) (see Fig. 3). They are organized by section.

1. **Merging all sequence annotations**: TSV files containing for each of the most abundant sequences of a locus: the identifier, the sequence and the annotation found by searching against all databases.
2. **Mapping**: BAM files containing mapping information of cleaned reads against the reference genome.
3. **Sequence Annotation**: BAM files containing mapping information of the most abundant sequence of each locus aligned against all databases.
4. **Prediction, functional and structural annotation of loci**: FASTA files of all loci identified and associated GFF3 files giving corresponding coordinates and annotations.
5. **Prediction, functional and structural annotation of miRNA**: Text file representing the secondary structures of potential miRNAs, FASTA file of potential miRNA sequences and GFF3 files of predictions and annotations of miRNAs and pre-miRNAs.
6. **Merging all sequences**: FASTA file containing cleaned sequences, TSV file containing the abundance table of each sequence for all samples, FASTA file containing low-abundant sequences (not taken into account in the analysis).
7. **Cleaning fastq files**: FASTQ files containing cleaned reads (after adaptor trimming, size and abundance filtering).
8. **Loci builder**: GFF3 file containing coordinates and annotations of loci all along the reference genome.

## 4 Experimental Design, Materials and Methods

### 4.1 CyHV-3 propagation onto CCB cells

A viral strain of CyHV-3 (n°197/ITT-V12) isolated from Italy and kindly provided by Istituto Zooprofilattico delle Venezie was inoculated on common carp brain (CCB) cells. The CCB cells were cultured in Dulbecco’s Modified Eagle’s Medium (DMEM, Sigma) supplemented with 10 % fetal bovine serum. The strain was passaged 26 times on CCB cells at 22 °C and 26 °C.

To examine the expression of microRNAs along a lytic cycle, 1 mL of infected cell culture supernatant was inoculated onto 24-h monolayers of CCB cells in T25 flasks (Falcon® 25 cm², Life Sciences), as detailed in Fig. 2. For the virus grown at 22 °C, the flasks were kept at 22 °C for 1 h, whereas for the virus grown at 26 °C, the flasks were kept at 26 °C for 30 min and then at 30 °C for another 30 min. This short step at 26 °C was designed to favour virus attachment to the cells before placing them at 30°C, which is known to be a non-permissive temperature [4]. Four T25 flasks were also inoculated with DMEM to serve as non-infected controls. After this one-hour incubation, four mL of complete DMEM medium were then added to the flasks, which were incubated at either 22 or 30 °C. Both cell monolayers and culture supernatants were harvested at different times post-inoculation: 1 h, 2 h, 4 h, 1 day, 2 days, 3 days, 6 days and 10 days. Four flasks were harvested at each collection time: 2 from 22 °C and 2 from 30 °C, to be used as duplicates. The supernatant was removed and frozen at −80 °C; the corresponding cell monolayers were trypsinised, rinsed with PBS, resuspended in 300 µL of lysis buffer (Nucleospin miRNA extraction kit, Macherey Nagel) and stored at −80 °C until RNA extraction. Non-infected controls (NIC) were harvested after 10 days, according to the same protocol.

### 4.2 Extraction of microRNAs, library preparation and sequencing

Small RNAs (miRNAs) were extracted from the cell pellets using the Nucleospin miRNA extraction kit (Macherey Nagel), following the manufacturer's instructions. Briefly, the cell lysates were thawed, incubated at room temperature for 5 min and transferred onto the purification columns. The bound miRNAs were washed and eluted in 100 µL (high yield) of 10 mM Tris-HCl, pH 8.5. Their purity was checked by spectrophotometry (Nanodrop 2100), and their integrity and size were verified using a BioAnalyzer 2100 (Agilent Technologies). The average size distribution ranged between 100 and 150 nucleotides, indicating that samples contained a majority of small RNAs. About 50 ng of small RNA were used to establish the RNA libraries of each sample using the TruSeq small RNA sample prep Kit (Illumina). Adapter ligation, reverse transcription and indexing were performed according to the manufacturer’s recommendations. The resulting cDNAs were run onto an acrylamide gel, and fragments of around 145 bp were recovered and used for sequencing. Samples were sequenced on 4 lanes (9 samples per lane) of a HiSeq2000 platform (Illumina) at Montpellier Genomix (Montpellier, France).

### 4.3 Analysis of miRNA sequences

Raw sequences were subjected to a first fast QC quality control, and all reads with a Phred score lower than 30 were excluded. They were then aligned against potential contaminants, such as bacteria, yeast, phages or mammals, with FastQ Screen. All reads that passed these controls were trimmed to remove the adaptor sequences using CutAdapt v1.4.1 [5] and filtered on length (min_length: 18bp, max_length: 26bp). The size distribution of the trimmed sequences was centered around 22-23 nt. Redundant reads were collapsed within each of the samples to eliminate redundancy, followed by the computation of unique read copy numbers. A total of 1,820,763 unique sequences were obtained from 376,502,679 reads. Only reads with a copy number greater than ten were retained.

They were then mapped against KHV-U reference genome (#DQ657948.1) or *Cyprinus carpio* genome assembly (#GCA_000951615.2) with Bowtie2 (v2.1.0, seed size of 16 (-L 16), 0 mismatch allowed in seed (-N 0), report 10 alignment per read (-k 10)) [6] and samtools (v0.1.19-44428cd) [7]. Reads mapping to a same locus in the reference genome were assembled into a longer region resulting in a potential pre-miRNA locus. Each locus was submitted to the annotation process and ncRNA prediction.

To annotate a locus, non-coding RNA public databases were used to assign one or more putative functions (miRBase (release 21) [8], eukaryotic-tRNAs from GtRNAdb (release 15) [9], Rfam (v. 11.0) [10], SILVA (v. 119) [11]. Bowtie2 [6] was used for mapping locus against these databases with a seed size of 16, allowing 0 mismatch in seed and reporting only 1 location.

Each locus then underwent a prediction step to determine whether it was a potential miRNA. For each candidate miRNA (annotated miRNA and orphan-annotated loci), the upstream and downstream flanking regions were extracted to consider their potential to form the expected pre-miRNA hairpin secondary structure. The same procedure as defined in Juanchich et al. [12] was applied.

## Limitations

It was originally planned to have three replicates at each stage of experimental infection, for the two temperatures. However, one of the three series was lost during the RNA extraction procedure, and only two replicates could be analysed for the expression of miRNAs, at both temperatures. The loss of the third replicate prevented from performing statistical tests regarding the kinetics of miRNA expression along the viral lytic cycle.

The small RNA population was diverse, encompassing miRNAs, siRNAs, piRNAs, but also fragments from other ncRNAs such as snoRNA, tRNA and rRNA fragments. Many small RNAs shared similar features, making it difficult to distinguish between them. This is a common limitation when studying non-model organisms, for which well curated, high-quality miRNAs do not exist.

## Ethics Statement

Authors have read and followed the ethical requirements for publication in Data in Brief. They confirm that the current work does not involve human subjects, animal experiments, or any data collected from social media platforms.

## Credit Author Statement

**OR:** methodology, formal analysis, software, writing original draft. **NNF:** investigation, writing original draft. **SH:** conceptualization, methodology, investigation. **CG:** methodology, formal analysis, software, writing original draft. **JCA:** conceptualization,funding acquisition, validation, writing original draft.

## Data Availability

Sequence Read ArchiveMicro-RNA expression data from common carp brain cells infected by Cyvirus cyprinidallo3 at permissive and non-permissive temperatures (Original data)
NCBICyprinid herpesvirus 3 genome and microtranscriptome sequencing (Original data)
